# Salubrinal protects cardiomyocytes against apoptosis in a rat myocardial infarction model via suppressing the dephosphorylation of eukaryotic translation initiation factor 2α

**DOI:** 10.3892/mmr.2015.3508

**Published:** 2015-03-18

**Authors:** RUI-JUN LI, KUN-LUN HE, XIN LI, LI-LI WANG, CHUN-LEI LIU, YUN-YUN HE

**Affiliations:** 1 Department of Cardiology, Chinese PLA General Hospital, Beijing 100853, P.R. China; 2 Beijing Institute of Pharmacology and Toxicology, Beijing 100850, P.R. China

**Keywords:** endoplasmic reticulum stress, eukaryotic translation initiation factor 2α, cardiomyocytes, apoptosis, myocardial infarction

## Abstract

The aim of the present study was to examine the role of eIF2α in cardiomyocyte apoptosis and evaluate the cardioprotective role of salubrinal in a rat myocardial infarction (MI) model. Rat left anterior descending coronary arteries were ligated and the classical proteins involved in the endoplasmic reticulum stress (ERS)-induced apoptotic pathway were analyzed using quantitative polymerase chain reaction and western blot analysis. Salubrinal was administered to the rats and cardiomyocyte apoptosis and infarct size were evaluated by a specific staining method. Compared with the sham surgery group, the rate of cardiomyocyte apoptosis in the MI group was increased with the development of the disease. It was also demonstrated that the mRNA and protein levels of GRP78, caspase-12, CHOP and the protein expression of p-eIF2α were increased in the MI group. Furthermore, the results showed that treatment with salubrinal can decrease cardiomyocyte apoptosis and infarct size by increasing eIF2α phosphorylation and decreasing the expression of caspase-12 and CHOP. The present study suggests that salubrinal protects against ER stress-induced rat cadiomyocyte apoptosis via suppressing the dephosphorylation of eIF2α in the ERS-associated pathway.

## Introduction

A large number of myocardial cells die after acute myocardial infarction (MI), which can result in ventricular remodeling and heart failure (1). Apoptosis is the predominant form of cardiomyocyte death, which occurs in acute or chronic MI (2,3). Therefore, greater understanding of the mechanism of apoptosis following MI may aid in the development of effective therapy to treat patients with MI.

Recent studies have shown that endoplasmic reticulum stress (ERS) is an important pathway in cell apoptosis in addition to the mitochondria pathway and death receptor pathway in heart failure (4,5). ERS can be caused by a number of disturbances, including hypoxia, nutritional deprivation, infection and drug toxicity (6). When a large amount of unfolded proteins accumulate in the ER, three ER receptors are disassociated with GRP78 and then unfolded protein response (UPR) is triggered (7). Two independent pathways mediated by ER transmembrane proteins are involved in the UPR protection mechanism. One pathway involves IRE1 and ATF6 restoring the unfolded or mis-folded protein to a correctly folded protein by inducing the expression of chaperone protein in the ER, which halts ERS and thus prevents cell apoptosis (8). The other involves the phosphorylation of eIF2α at serine residue 51 by pancreatic endoplasmic reticulum eIF2α kinase (PERK) and phosphorylated eIF2α decreases the unfolded protein level by preventing protein synthesis (9,10). When these two pathways were inhibited, the apoptosis-related proteins, such as caspase-12, GADD/CHOP and ASK1/JNK, were activated and ERS-induced apoptosis was then triggered (9). The ER-related apoptotic pathway has been reported to be one of mechanisms underlying the induction of MI injury in cardiomyocytes (11).

Salubrinal is a small molecular compound that can inhibit the dephosphorylation and maintain the phosphorylation state of eIF2α, resulting in the suppression of protein translation and a decrease in protein synthesis to maintain homestasis in the ER (12,13). The protective effect produced by salubrinal has been verified in a number of studies (14–16). Salubrinal protects against cell death, which was mediated by tunicamycin-induced ERS at certain concentrations in the PC-12 rat cell line (17). Gasparetto *et al* (18) found that salubrinal can be used to reduce nephrotoxicity induced by cyclosporine-mediated ERS in rats. In addition, salubrinal has also been used for the treatment of urinary system diseases (19) and diabetes (20). Recently, Liu *et al* (21) have suggested that salubrinal can protect against tunicamycin- and hypoxia-induced cardiomyocyte apoptosis via the PERK-eIF2α signaling pathway. In the heart failure rat model, salubrinal treatment reduced apoptosis and increased the levels of eIF2α and caspase-12 (16). However, to the best of our knowledge, there has been no study investigating the effects of salubrinol in MI.

Thus, in the present study, ERS was observed in a rat MI model. Salubrinal was used as an *in vivo* treatment and eIF2α phosphorylation was detected and its role in MI was evaluated.

## Materials and methods

All studies were approved by the Chinese PLA General Hospital Ethics Committee and performed in accordance with the ethical standards of the Authorization for Practicals on Animals at our hospital. All the experiments were conducted in accordance with the Guide for the Care and Use of Laboratory Animals and approved by the ethical committee of Academy of Military Medical Science.

### MI rat model

Male Wistar rats weighing 220–260 g (Animal Center affiliated to Academy of Military Medical Science, Beijing, China) were randomly divided into the MI group (n=75) and sham group (n=75). Each group was further divided into five subgroups (15 rats per group), which were anaesthetized and sacrificed at 1, 3, 6, 12 and 24 h, respectively. In the salubrinal-treated experiments, an additional 60 rats were equally and randomly divided into the sham group, MI group and salubrinal-treated group (n=20). Heart tissue analysis in the sham group, MI group and salubrinal group was performed after 24 h administration.

Rats were anaesthetized with intraperitoneal (ip) injection of 2% pentobarbital natrium (40 mg/kg, Shanghai Second Chemical Reagent Company, Shanghai, China). The rats were placed in the supine position. A left thoracotomy was performed and the left anterior descending (LAD) coronary artery was ligated at the root of the left coronary artery as previously reported (22). Successful coronary occlusion was confirmed by a typical S-T segment elevation on the electrocardiogram, whitened myocardial tissue and decreased regional myocardial velocity, as determined by magnetic resonance imaging, which was performed on a Picker 1.5-T scanner (Picker International, Highland Heights, OH, USA), as described previously (23). No LAD coronary artery ligation was performed in the sham group. Furthermore, in the salubrinal treated group, rats received ip injection of salubrinal (1 mg/kg body weight, Sigma-Aldrich, St. Louis, MO, USA) for 30 min prior to LAD ligation. Salubrinal was dissolved in dimethy sulfoxide (Amresco, Solon, OH, USA) and then in saline. The MI group with ip injection of equal volume of DMSO and saline was set as the control.

### Hematoxylin and eosin (H&E), terminal deoxynucleotidyl transferase-mediated dUTP nick end labeling (TUNEL) and triphenyltetrazolium chloride (TTC) staining

The heart tissues from each group were fixed in 10% neutral formaldehyde and then stained with H&E (Beijing Chemical Reagent Company, Beijing, China). Images were captured using a microscope (BX51; Olympus Corporation, Tokyo, Japan).

The pretreated heart tissue slices were processed with TUNEL staining according to the manufacturer’s instructions (Roche, Basel, Switzerland). The slices were analyzed under a light microscope with a magnification of ×400. Five non-overlapping fields were randomly selected and the cells with brown particles observed in the nucleus were recognized as positive cells. The following equation was used for the apoptotic index (AI) calculation: AI=(Positive cell numbers in one field/Total cell numbers in one field) × 100.

The hearts was rapidly removed and cooled in ice-cold saline for 10 min. Left ventricle (2 mm) was cut and immersed in 1% TTC (Sigma-Aldrich) at 37°C for 30 min, and then transferred to 4% paraformaldehyde in 0.1 M PBS (pH 7.4) for 24 h fixation. Normal hearts were red while MI hearts were white. The heart slices were photographed and analyzed with Image-Pro Plus 6.0 (Media Cybernetics, Inc., Rockville, MD, USA).

### Lactate dehy drogenase (LDH), creatine kinase (CK) and superoxide dismutase (SOD) activity

LDH, CK and SOD activity in myocardial tissue were measured using LDH, CK and SOD Detection kits (Nanjing Jiancheng Bioengineering Institute, Nanjing, China) according to the manufacturer’s instructions.

### Reverse transcription-quantitative polymerase chain reaction

Total RNA was extracted from myocardial tissue by TRIzol reagent (Invitrogen Life Technologies, Carlsbad, CA, USA) and DNA was removed by DNase (Sigma-Aldrich). The reverse transcription reaction was performed according to the manufacturer’s instructions. The GRP78, caspase-12 and CHOP specific sequences were amplified during 45 cycles of 20 sec denaturing at 95°C, 25 sec annealing at 59°C, and 30 sec extension at 72°C, with the primers listed in Table I. GAPDH was used as an internal control and the 2^−ΔΔCt^ method was used to calculate expression levels.

### Western blot analysis

The frozen myocardial tissues were lysed in radioimmunoprecipitation cell lysis solution (Beyotime Biotechnology, Shanghai, China), followed by high speed centrifugation (13,000 × g, 5 min) and β-cyanoalanine quantification. Cellular protein was separated by electrophoresis on SDS-PAGE gel and then transferred onto polyvinylidene difluoride membrane (Merck Millipore, Darmstadt, Germany). After blocking, the blots were incubated with antibodies against GRP78 (N-20) (cat. no. sc-1050; 1/600; Santa Cruz Biotechnology Inc., Santa Cruz, CA, USA), p-eIF2α (Ser49) (cat. no. sc-293100; 1/200; Santa Cruz Biotechnology Inc.), caspase-12 (A-14) (cat. no. sc-12395; 1/400; Santa Cruz Biotechnology Inc.) and GADD153 (F-168) (cat. no. sc-575; 1/500; Santa Cruz Biotechnology Inc.) at 4°C overnight. The eIF2α (FL-315) (cat. no. sc-11386; 1/500; Santa Cruz Biotechnology Inc.) and actin (C-11) (cat. no. sc-1615; 1/500; Santa Cruz Biotechnology Inc.) were used as loading controls. The appropriate horseradish peroxidase-conjugated secondary antibodies were added and the samples were incubated for 1 h at room temperature (1:6,000 dilution for p-eIF2a and caspase-12 and 1:10,000 dilution for GRP78, eIF2a, GADD153 and actin). The protein bands were detected with SuperSignal Ultra Chemiluminescent Substrate (Pierce, Rockford, IL, USA) on X-ray films (Koda).

### Statistical analysis

Statistical analysis was performed by SPSS11.5 software (SPSS Inc., Chicago, IL, USA). The data are presented as the mean ± standard deviation. One way-analysis of variance was used to examine the differences between three or more groups. P<0.05 was considered to indicate a statistically significant difference.

## Results

### UPR of ER responds to MI

**Figure 1:**
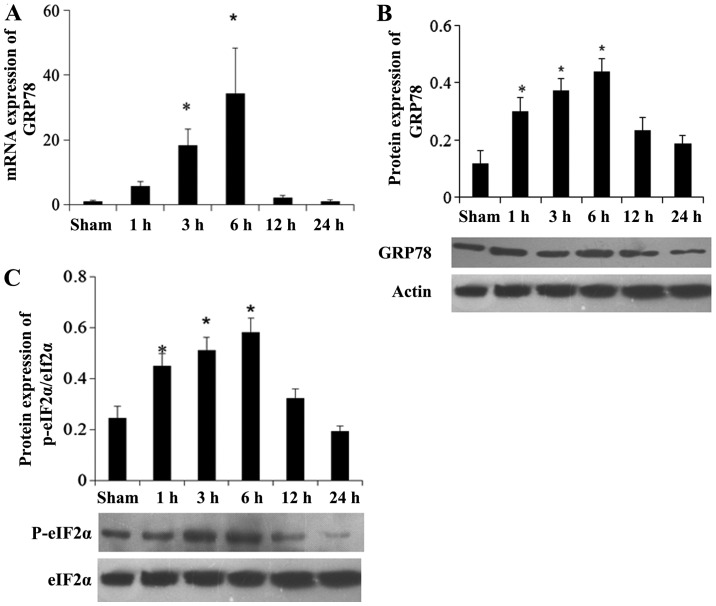
Myocardial infarction activates the unfolded protein response by inducing GRP78 and p-eIF2α expression. (A) Relative mRNA levels of endoplasmic reticulum stress-associated gene GRP78 were measured by reverse transcription-quantitative polymerase chain reaction. Western blot analysis of (B) GRP78 and (C) p-eIF2α is shown. Actin and eIF2α were used as internal controls. The representative images from three independent experiments were presented. Data are presented as the mean ± standard deviation (^*^P<0.05 vs. sham). p-eIF2α, phosphorylated-eukaryotic translation initiation factor 2α.

After successful establishment of the MI rat model, RT-qPCR and western blot analysis were used to analyze the expression of GRP78 in the myocardial tissues. Compared with the sham group, the mRNA expression level of GRP78 in the MI group was increased during the early period after MI and decreased after 6 h of MI (Fig. 1A). Furthermore, the western blot analysis results showed that no significant difference in the GRP78 protein level was identified among the different time points in the sham group; thus, the GRP78 protein level at the 24 h time point in sham group was used as a reference. Compared with the sham group, GRP78 protein in the MI group was expressed 1 h following the establishment of MI and reached a peak after 6 h (Fig. 1B). A similar outcome was demonstrated via immunohistochemistry (data not shown).

Furthermore, the phosphorylation level of eIF2α was detected by western blot analysis and immunohistochemistry. Western blot analysis results showed that p-eIF2α protein expression was increased and reached a peak 6 h after MI (Fig. 1C), indicating that UPR was induced at early stages following establishment of MI. Immunohistochemistry experiments showed similar results (data not shown).

### ER stress-induced apoptosis pathway is activated after MI

**Figure 2:**
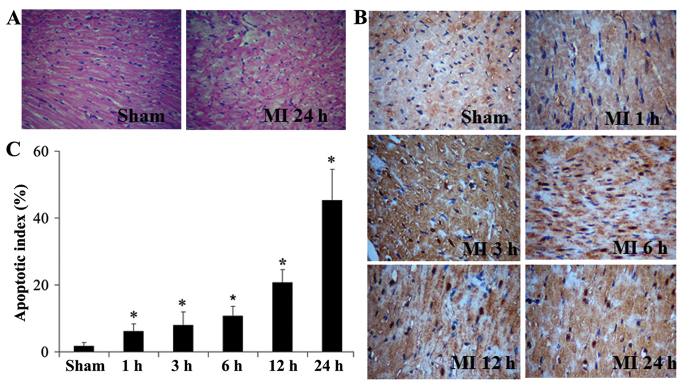
MI induces cardiomyocyte apoptosis. The morphological changes were observed by H&E staining and apoptosis of cardiomyocytes was examined by using TUNEL after MI. (A) Morphological changes were determined 24 h after MI and sham operation by H&E staining. (B) Cardiomyocyte apoptosis was measured by TUNEL at 1, 3, 6, 18, 24 h after MI. (Original magnification, ×400). (C) The figure shows the apoptotic index of cardiomyocytes in different groups at different times (mean ± standard deviation). MI, myocardial infarction; TUNEL, terminal deoxynucleotidyl transferase-mediated dUTP nick end labeling; H&E, hematoxylin and eosin; eIF2α, eukaryotic translation initiation factor 2α.

According to the H&E staining results, myocardial injury was observed in the MI group, while no significant difference was observed among different time points in the sham group (Fig. 2A). TUNEL staining also showed no difference in AI among different time points in the sham group (P>0.05). However, in the MI group, AI was increased with time and was significantly higher compared with that in the sham group following MI (P<0.05, Fig. 2B and C).

**Figure 3:**
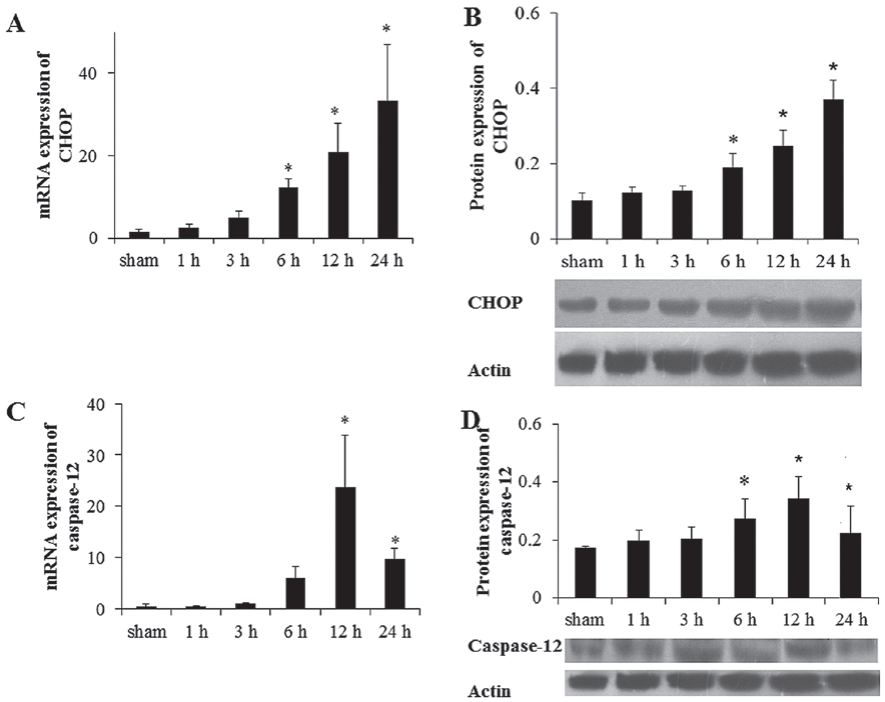
Prolonged MI activated the ER stress-induced apoptosis pathway. Relative mRNA levels of ER stress-associated genes (A) CHOP and (B) cas-pase-12 were measured by reverse transcription-quantitative polymerase chain reaction. Western blot analysis of (C) CHOP and (D) caspase-12 are shown. Actin was used as an internal control. The representative images from three independent experiments are presented. Data are presented as the mean ± standard deviation (^*^P<0.05 vs. sham). MI, myocardial infarction; ER, endoplasmic reticulum.

Subsequently, the mRNA and protein expression levels of CHOP and caspase-12 protein, which were involved in the apoptotic pathway induced by ER were investigated. Compared with the sham group, the mRNA level of CHOP and caspase-12 were increased 6 h after MI and reached a peak at 24 and 12 h after MI, respectively (Fig. 3A and B). Compared with the sham group, the CHOP protein level was increased significantly at 6 h after MI and reached a peak at 24 h (P<0.05; Fig. 3C). The protein level of caspase-12 was increased 6 h after MI and declined significantly at 24 h after MI (Fig. 3D). Similar results were detected via immunohistochemistry (P<0.05; data not shown).

### Salubrinal increases the phosphorylation level of eIF2α and decreases the protein level of CHOP and caspase-12

**Figure 4:**
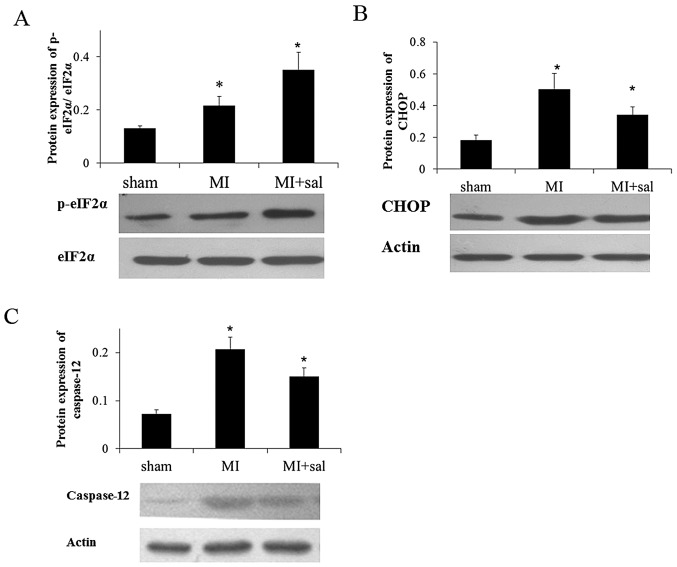
Salubrinal treatment increases the p-eIF2α protein level and decreases the protein level of CHOP and caspase-12 after MI. Western blot analysis of (A) p-eIF2α, (B) CHOP and (C) caspase-12 is shown. Actin and eIF2α were used as an internal control. The representative images from three independent experiments were presented. Data are presented as the mean ± standard deviation (^*^P<0.05 vs. sham). MI, myocardial infarction; p-eIF2α, phosphorylated-eukaryotic translation initiation factor 2α.

Salubrinal was administered to MI rats. In the salubrinal treated group, the p-eIF2α expression level was increased significantly compared with the MI group, which was verified by western blot analysis (P<0.05) (Fig. 4A). Furthermore, the protein levels of CHOP and caspase-12 were decreased significantly in the salubrinal treated group compared with the MI group (P<0.05) (Fig. 4B and C). Immunohistochemistry experiments demonstrated the same results (data not shown).

### Ischemic damage induced by MI is alleviated by suppression of eIF2α phosphorylation

**Figure 5:**
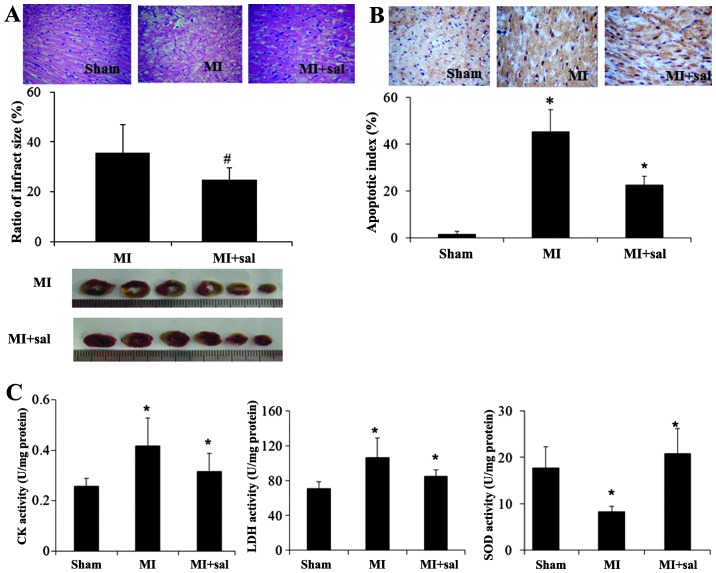
Ischemic damage induced by MI was decreased by suppressing eIF2α. The effect of salubrinal (sal) treatment on the myocardium was observed by (A) hematoxylin-eosin staining and triphenyltetrazolium chloride staining, (B) terminal deoxynucleotidyl transferase-mediated dUTP nick end labeling (original magnification, ×400), and (C) myocardial enzyme detection (CK, LDH, SOD). The representative images from three independent experiments were presented. Data were presented as mean ± standard deviation (^*^P<0.05 vs. sham; ^#^P<0.05 vs. MI). MI, myocardial infarction; eIF2α, eukaryotic translation initiation factor 2α; LDH, lactate dehy drogenase; CK, creatine kinase; SOD, superoxide dismutase.

According to H&E staining, the myocardial damage in the salubrinal treated group was alleviated compared with the MI group. The MI size assessed by the TTC staining was decreased significantly in the salubrinal treated group compared with the MI group (P<0.05, Fig. 5A). TUNEL staining showed similar results (Fig. 5B). The activities of CK and LDH were decreased and SOD was increased in the salubrinal treated group, compared with the MI group (Fig. 5C).

## Discussion

To the best of our knowledge, the present study is the first to demonstrate that salubrinal protects cardiomyocytes against apoptosis induced by MI. It was shown that administration of salubrinal protected cardiomyocytes from injury induced by LAD ligation in a rat model. Enhanced eIF2α phosphorylation and decreased MI size were observed in the salubrinal treated group. These findings indicated that the protective effect of salubrinal in the rat MI model was mediated by suppressing the dephosphorylation of eIF2α.

When ER dysfunction occurs, GRP78 is released and interacts with unfolded or mis-folded protein to prevent protein accumulation in the ER. As GRP78 is induced in response to ER dysfunction, it was used as a marker to indicate the events of ERS and UPR (24). In the present study, the mRNA level of GRP78 increased significantly and reached a peak at 6 h, and the GRP78 protein was expressed 1 h after MI and also reached a peak at 6 h in the MI group. These results suggested that UPR was induced early after MI. The results were consistent with a previous study, which demonstrated that GRP48 expression was increased in cardiac myocytes near the MI (25). In addition, p-eIF2α protein expression was increased and reached a peak 6 h after MI. The increase in p-eIF2α expression was also observed following heart failure (16). All the results showed that the ER was activated after MI.

When ER stress occurred, caspase-12 was activated, which mediates ER-specific apoptosis (26–28). Therefore, caspase-12 can be recognized as a specific marker of ERS-induced apoptosis. It was demonstrated that the expression of caspase-12 was elevated 6 h after MI and gradually increased with time. These results indicated that UPR was induced to respond to ERS during the early stage after MI, in which no caspase-12 was detected. However, with the processing of ERS, caspase-12 was activated and cell death was observed. Similar results were shown in a previous study where the protein expression of caspase-12 was increased in a heart failure rat model, which was induced by MI (16).

CHOP is also termed GADD153, the activation of which is one of the ER apoptosis pathways (29). Studies have demonstrated that CHOP can be used as a specific marker in ERS induced apoptosis (30). In the present study, it was demonstrated that the protein level of CHOP began to increase 6 h after MI and reached a peak at 24 h. No CHOP expression was observed during the early stages of MI as UPR was induced. With the development of MI, CHOP was activated and apoptosis was triggered.

When MI model rats were treated with salubrinal, infarct size was reduced and apoptosis was alleviated. Similarly, the activities of LDH and CK, which have high specificity for cardiac injury and infarction, were decreased following administration of salubrinal. Antioxidant enzyme SOD activity was decreased after MI and increased after salubrinal treatment, which has been reported to be impaired in MI (31). The results showed that salubrinal alleviated the ischemic damage. In salubrinal treatment, expression levels of CHOP and caspase-12 were decreased. In addition, the phosphorylation level of eIF2α was increased following salubrinal treatment. It has been reported that salubrinal could inhibit the dephosphorylation of eIF2α (32). In HK-2 human renal proximal tubular cells exposed to cadmium chloride, apoptosis was induced and salubrinal could reduce the expression of CHOP expression (33). In heart failure, caspase-12 was increased in apoptotic cardiac myocytes and significantly decreased after salubrinal treatment (16). In heart failure, the phosphorylation of eIF2α was decreased and increased further after salubrinal treatment (16,34). Thus, salubrinal could protect cardiac myocytes against ERS apoptosis after MI.

In conclusion, using the rat LAD ligation model, it was found that ERS was induced in the cardiomyocyte apoptosis following MI. UPR was found to maintain ER homestasis at the early stages of MI and then the apoptosis related pathway was activated. eIF2α may therefore be a candidate gene for an MI therapeutic target as the eIF2α inhibitor salubrinal in the rat model can alleviate the damage of MI. However, these results require further confirmation.

## Figures and Tables

**Table I:** Sequence of the primers.

Primer name	Forward (5′-3′)	Reverse (5′-3′)
Caspase-12	CTCTTCATTTCCAAACTCGTTGACT	GGGCATCTGGGTCAGTTCAC
GADD153/CHOP	AGGAGGTCCTGTCCTCAGATGA	ATGTGCGTGTGACCTCTGTTG
GRP78	TGACCAAAACCGCCTGACA	TCTCCAATCTGGTTCTTGAGAGAA
GAPDH	GGTGAAGGTCGGTGTGAACG	CTCGCTCCTGGAAGATGGTG
